# Electrocardiographic and electrophysiological characteristics of idiopathic ventricular arrhythmias originating from the vicinity of tricuspid annulus

**DOI:** 10.1038/s41598-021-88036-7

**Published:** 2021-04-21

**Authors:** Manli Yu, Liangliang Hou, Hang Yu, Junwei Ge, Pan Li, Zhifu Guo, Xinmiao Huang, Xianxian Zhao, Jiang Cao, Songqun Huang

**Affiliations:** 1 Department of Cardiovasology, Changhai Hospital, Second Military Medical University, 168 Changhai Road, Shanghai, 200433 China; 2 Department of Emergency, Changhai Hospital, Second Military Medical University, 168 Changhai Road, Shanghai, 200433 China; 3 Department of Cardiovasology, Shanghai Seventh People’s Hospital, Shanghai, 200433 China

**Keywords:** Cardiology, Interventional cardiology

## Abstract

Electrocardiographic and electrophysiological characteristics of VAs originating from the vicinity of the TA are not fully understood. Hence, 104 patients (mean age 52.6 ± 17.9 years; 62 male) with VAs originating from the vicinity of the TA were enrolled. After electrophysiological evaluation and ablation, data were compared among those patients. The ECGs and the correction of the ECGs based on the long axis of the heart calculated from the chest X-Ray were also analyzed. VAs originating from the vicinity of TA had distinctive ECG characteristics that were useful for identifying the precise origin. Our localization algorithm adjusted by the angle between the cardiac long axis and the horizon was found to be accurate in predicting the exact ablation site in 92.3% (n = 96) cases. Logistic regression analysis showed fractionated electrograms, the magnitudes of the local atrial electrograms and a/V ratio were critical factors for successful ablation. Among the 104 patients with VAs, complete elimination could be achieved by RFCA in 96 patients (success rate 92.3%) during a follow-up period of 35.2 ± 19.6 months. This study suggests that the ablation site could be localized by ECG analysis adjusted by the angle between the cardiac long axis and the horizon. Fractionated electrograms, the magnitudes of the local atrial electrograms and a/V ratio were demonstrated to be critical factors for successful ablation.

## Introduction

Idiopathic Ventricular arrhythmias (VAs) with a focal mechanism including premature ventricular contractions (PVCs) and idiopathic ventricular tachycardias (IVTs) may occur in patients with or without structural heart disease^1–4^. The right ventricular outflow tract (RVOT) is the most common origin location of idiopathic VAs and can be easily eliminated by radiofrequency catheter ablation (RFCA)^5,6^. However, VAs originating from the vicinity of the tricuspid annulus (TA) have been reported to be challenging to successfully ablate because of the limited value of the ECG to predict the site of exact origin around the TA and uncertainty of the ablation target^7,8^. Since limited published data was documented, the purpose of this study was to analyze electrocardiographic and electrophysiological characteristics as well as the outcome of catheter ablation for such VAs originating from the vicinity of TA. We also reported a new localization algorithm that can help us to identify the ablation sites of VAs from the vicinity of TA before mapping.

## Materials and methods

### Study populations

There were 1037 consecutive patients with idiopathic ventricular arrhythmias who underwent RFCA at our institution between January 2009 and August 2018, among whom 104 (10.0%) patients (62 men and 42 women; age 52.6 ± 17.9 years) with VAs originating from the vicinity of TA were enrolled and followed up. Of 104 patients, 94 had frequent PVCs, and 10 of 104 manifested both PVCs and IVT. Baseline characteristics including age, sex, PVCs burden, duration of PVCs, 12-lead ECG morphology of PVCs, and right ventricular dimension in echocardiography were recorded. Patients with arrhythmogenic right ventricular cardiomyopathy (ARVC) or other structural heart disease were excluded by case history, physical examination, ECG, echocardiography, electroanatomical mapping and in some patients by cardiac magnetic resonance. All antiarrhythmic drugs were discontinued for at least 5 half-lives before RFCA. This study was approved by the Ethics Committee of the First Affiliated Hospital of the Second Military Medical University and was performed in accordance with the latest vision of the Declaration of Helsinki. All patients signed the form of informed consent before the study was initialized.

### Electrophysiologic study

For comparison, ECGs were analyzed from 104 consecutive patients with idiopathic VAs arising from the vicinity of TA. Relevant ECG data known to differentiate between VAs originating from the septal portion and the free wall of TA, such as QRS pattern and precordial transition zone were collected for analysis^9^.

### Definition of TA location for VAs origin

**Figure 1:**
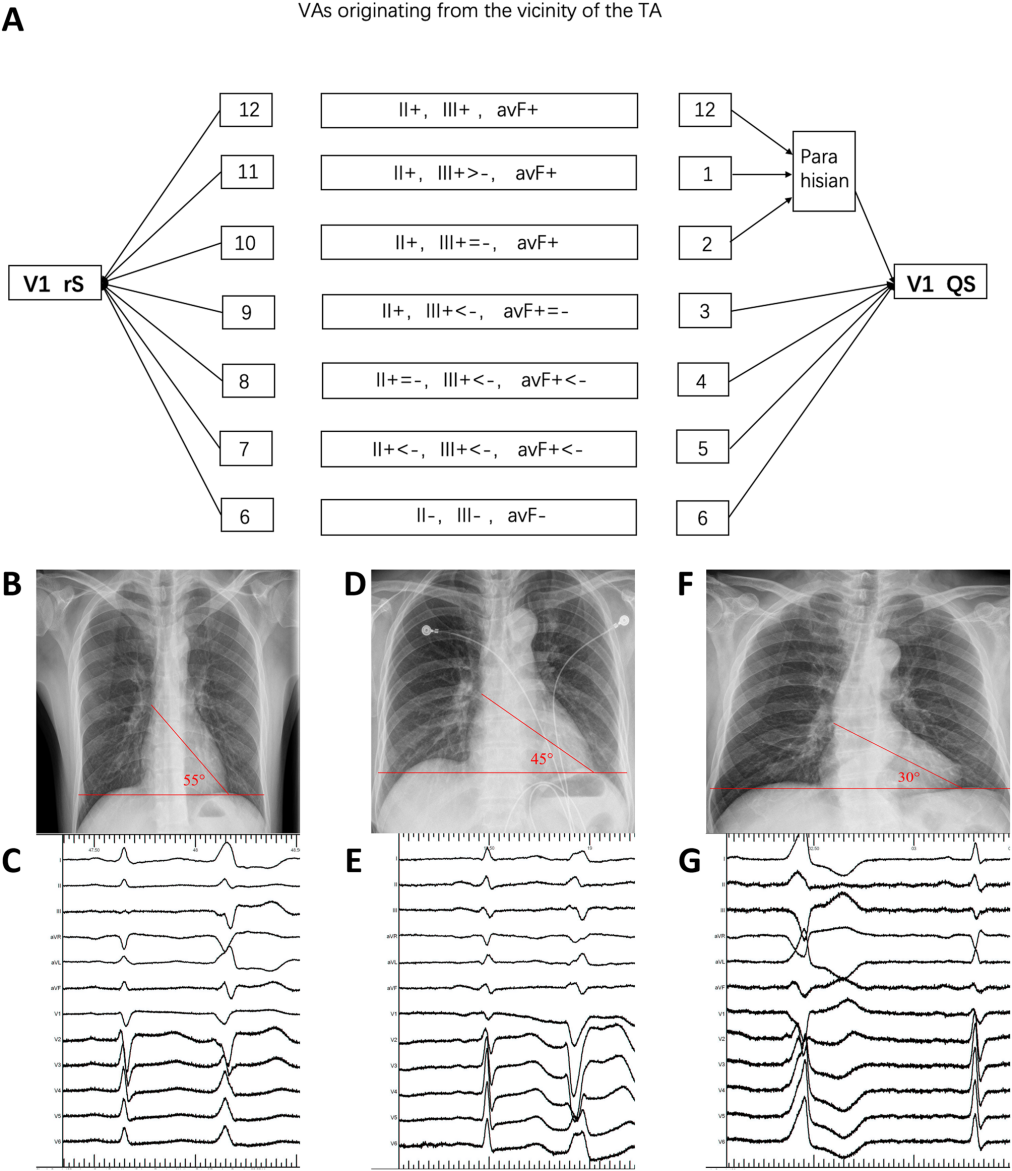
(**A**) This ECG algorithm was a proposed analysis of the frontal plane leads and was later adjusted by the long axis of the heart from the chest X-ray. The algorithm is used to localize the ablation sites of VAs from the vicinity of TA (“ + ” means a positive QRS polarity, “ − ” means a negative QRS polarity) .Degree of Angle*(baseline:45°)increase per 5°,location decrease 0.5 o’clock; decrease per 5°,location elevate 0.5 o’clock. (**C**, **E**, **G**) showed 12-lead ECG of PVCs in three patients. They all showed “aVF +  =  − ”. When adjusted by chest X-ray (**B**, **D**, **F**) the ablation target were located in para-hisian, 3 o’clock, 4 o’clock, respectively.

VAs were considered to arise from the different portions of TA based on the following criteria. First, the catheter tip demonstrated the successful ablation site at the characteristic location of the TA when viewed in the right anterior oblique (RAO) and left anterior oblique (LAO) fluoroscopic views. Second, the amplitudes of the atrial and ventricular electrograms were > 0.03 mV and > 0.35 mV at the ablation site, respectively. In the LAO view, TA was viewed as a clock face and the predicted ablation sites of VAs were analyzed according to our new algorithm (Fig. 1). The exact location was further confirmed by three-dimensional electromagnetic anatomy.

### Mapping and RFCA

**Figure 2:**
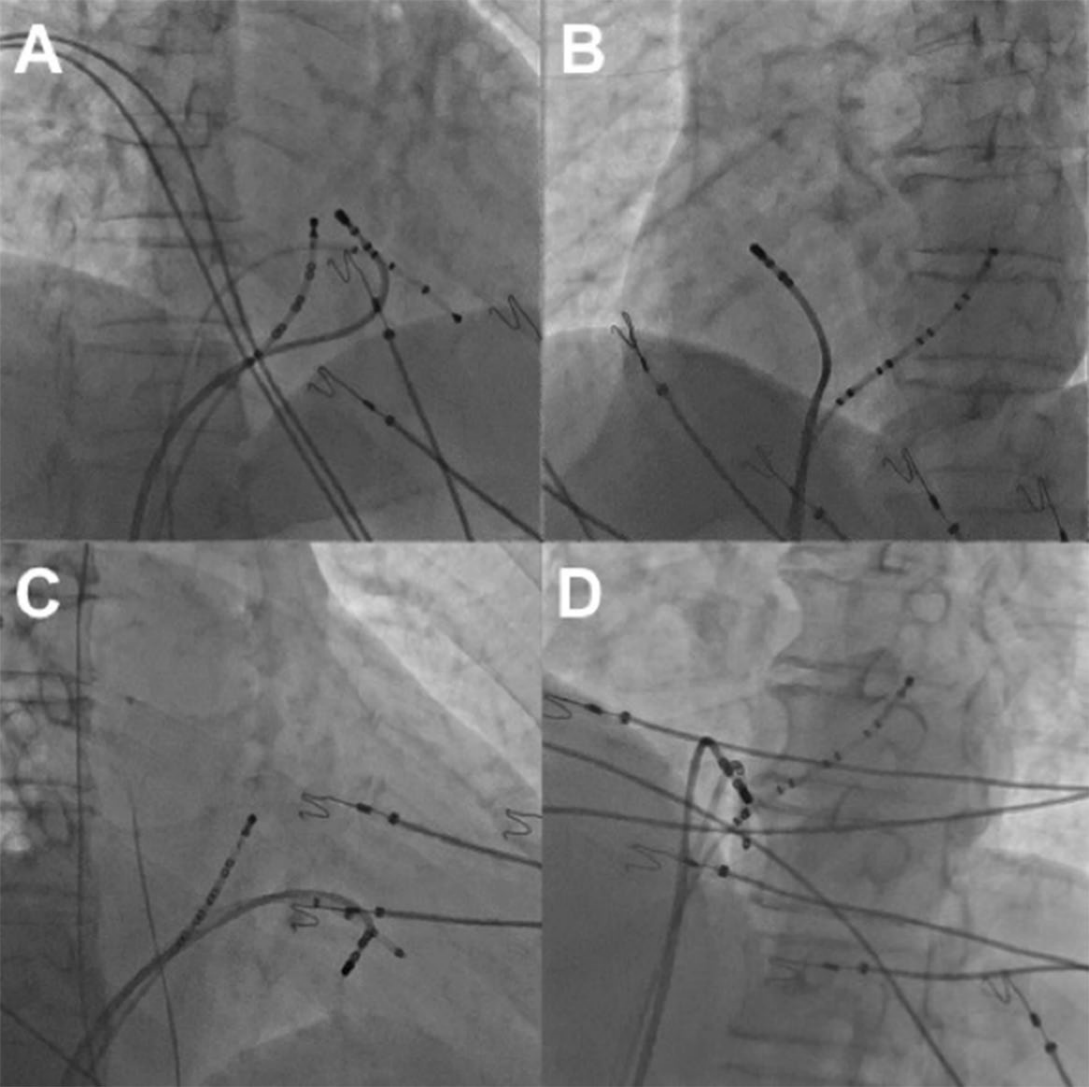
Successful ablation sites utilizing the reversed U-curve ablation technique (**A**) Right anterior oblique (RAO 30°) radiographic views of site at 11 o’clock; (**B**) Left anterior oblique (LAO 45°) radiographic views of site at 11 o’clock; (**C**) Right anterior oblique (RAO 30°) radiographic views of site at 5 o’clock; (**D**) Left anterior oblique (LAO 45°) radiographic views of site at 5 o’clock.

The RFCA was performed when frequent PVCs were present or ECGs of VT were recorded before procedure. Programmed electrical stimulation was performed to induce ventricular tachycardia using up to three ventricular extrastimuli at three drive cycle lengths from two right ventricular sites. Isoproterenol was used to induce VAs if necessary. An 8-F ablation catheter with a 4-mm distal electrode (Biosense Webster, Diamond Bar, California, USA) was used for mapping and ablation. An electroanatomic mapping system (Nav-X, St.JudeMedical, St. Paul, Minnesota) was used to guide mapping for all patients. The target site for RFCA was determined by activation mapping (earliest local activation time preceding the earliest surface QRS by ≥ 15 ms) in patients with frequent PVCs/sustained IVT, and by pace mapping (≥ 11/12–lead concordance of major and minor deflections between the pace map and the clinical PVCs) in those with infrequent arrhythmia. Bipolar pacing was performed at an output just greater than the diastolic threshold from the distal electrode pair (with the distal electrode as the cathode) during sinus rhythm. Besides those, magnitudes of atrial electrograms (a) and ventricular electrograms (V) as well as fractionated electrograms were also recorded while mapping. After the target site was located, ablation was performed using a maximum power of 20–50 W and maximum temperature of 55 °C. The “reversed U-curve” ablation technique was used if the conventional method (catheter was put directly near the tricuspid annulus and sometimes above the tricuspid valve) failed to eliminate PVCs. Ablation catheter was inserted in right ventricle and reversely fixed within the space between tricuspid valve and ventricular wall as demonstrated in Fig. 2. A 3.5-mm irrigated-tip electrode (Biosense Webster, Diamond Bar, California, USA) was used if necessary. A Swartz introducer sheath was used when the ablation catheter alone could not achieve enough contact. Mapping and ablation were applied in the non-coronary sinus, right coronary sinus, left ventricular outflow tract or the vicinity of mitral annulus in some patients, if VAs originating from the septal region could not be ablated in the vicinity of the TA. Patients were observed for thirty minutes following final ablation. Acute success was defined as the absence of PVCs or induced VTs during a thirty-minute observation after final ablation.

### Follow-up

After the procedure, antiarrhythmic drugs were discontinued if the ablation procedure was acutely successful. Patients were evaluated every 3 months in an outpatient clinic for 12–24 months and 24 h Holter monitoring was performed 3 months after the ablation. Whenever the patient had symptoms suggestive of recurrence of VAs, ECG and 24-h ECG monitoring were performed.

### Statistical analysis

Continuous variables are given as mean ± SD, and categorical variables are given as number (percentage). Student t test was used to compare continuous variables, whereas comparisons of categorical data were performed with the χ^2^ test or Fisher exact test, as appropriate. Logistics-regression analysis and Cox-regression analysis were used to examine the predictors of ablation failure and early recurrence. *P* < 0.05 was considered statistically significant. Statistical calculations were performed using SPSS (version 18.0, SPSS Inc, Chicago).

## Results

### Patient characteristics and ECG analysis

The study population consisted of 62 men and 42 women (age 52.6 ± 17.9 years, range 18–82 years) with symptomatic VAs. The medical history was significant for hypertension in 32 patients, diabetes in 10 patients, and coronary artery disease (no ischemia evident prior to the ablation procedure) in 10 patients. No structural heart disease was evident by echocardiography in all subjects. Basic clinical data are presented in Table 1.

**Table 1:** Characteristics of the study population.

	Patients (N = 104)
Age (years)	52.6 ± 17.9
Male sex	62/104(59.6%)
BMI	23.8 ± 3.3
**Concomitant disease**	
Hypertension	32/104 (30.8%)
Diabetes	10/104 (19.2%)
Coronary artery disease	10/104 (19.2%)
PVCs burden	18,463.6 ± 8425.2 (16.9 ± 8.1%)
**Cardiac ultrasound**	
LVEDD (mm)	51.4 ± 10.6
LVEF (%)	60.7 ± 3.2
RVEDD (mm)	23.1 ± 7.2
IVST (cm)	0.98 ± 0.12
**QRS morphology of PVCs/VT**	
QRS duration (ms)	133.3 ± 13.1
“Notching” in the inferior wall leads	35/104 (33.7%)
Precordial transition zone score	3.0 ± 0.7
Procedure duration (min)	117.4 ± 52.9
Fluoroscopy time (min)	11.2 ± 8.3

Values are given as mean ± SD (range) or n (%) unless otherwise indicated.

*LVEDD* Left ventricular end-diastolic diameter, *LVEF* Left ventricular ejection fraction, *RVEDD* Right ventricular end-diastolic diameter, *IVST* Interventricular septal thickness, Precordial transition zone was defined as the position of the precordial leads in which the amplitudes of the R and S waves were equal.

**Figure 3:**
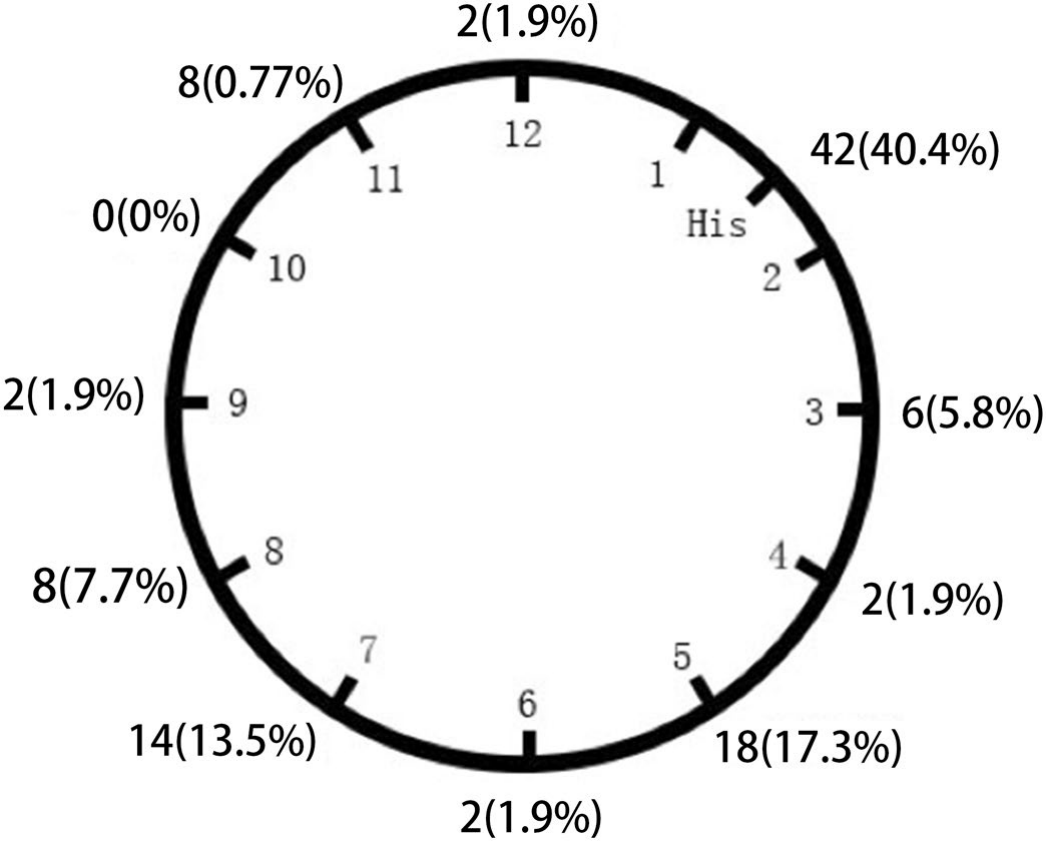
Schematics indicating the distribution of the origin of VAs originating from the vicinity of TA (the site of successful ablation or speculative ablation target).

Among the 104 patients with VAs, complete elimination could be achieved by RFCA in 96 patients (success rate 92.3%) during a follow-up period of 35.2 ± 19.6 months. Seventy VAs (67.3%) originated from the septal portion of the TA and the remaining 34 (32.7%) from the free wall portion of the annulus (Table 2). In the LAO view, TA was viewed as a clock face and schematic indicated the distribution of the origins of VAs originating from the vicinity of TA in Fig. 3.

**Table 2:** Clinical and ECG characteristics of VAs originating from different portions.

	Septal (N = 70)	Free wall (N = 34)	*P* value
Age (years)	59.9 ± 14.8	37.4 ± 14.0	0.001
Male sex	44/70 (62.9%)	18/34 (52.9%)	0.334
BMI	24.5 ± 3.3	22.2 ± 2.6	0.001
PVCs burden	16,558.2 ± 7376.4	18,846.7 ± 8349.1	NS
**QRS morphology of PVCs/VT**			
QRS duration	125.3 ± 5.8	149.7 ± 7.2	0.001
“Notching” in the limb leads	17/70 (24.3%)	18/34 (52.9%)	0.004
Precordial transition zone score	2.8 ± 0.70	3.5 ± 0.6	0.001
Procedure duration (min)	128.8 ± 50.9	111.7 ± 53.6	0.03
Fluoroscopy time (min)	11.9 ± 9.1	11.1 ± 10.8	0.97

### ECG analysis of VAs arising from the TA

**Figure 4:**
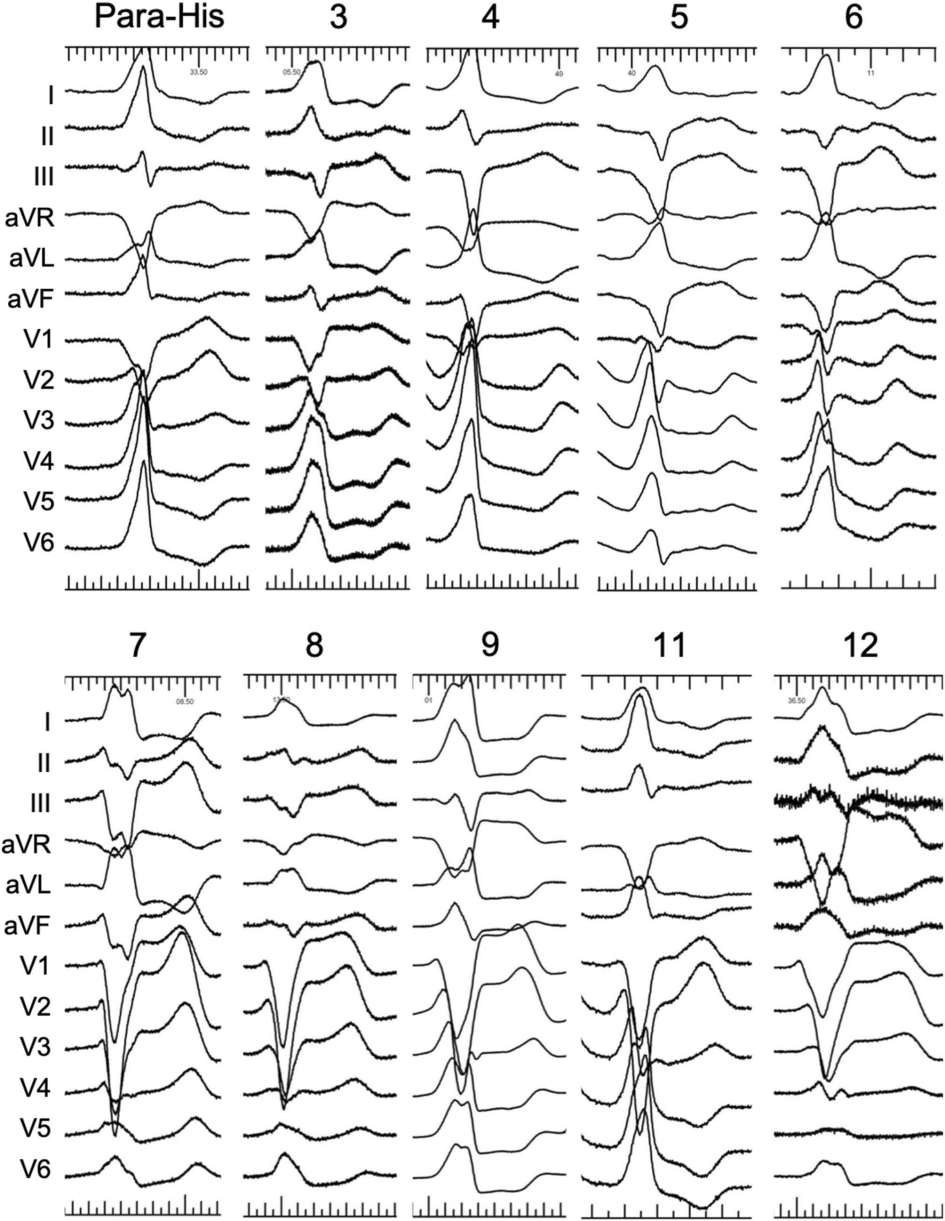
Typical twelve-lead QRS morphology of PVCs/VT from different regions.

QRS morphology of VAs by surface ECG and clinical characteristics in septal and free wall portions of TA are presented in Table 2. In all patients with VAs originating from the vicinity of TA, their QRS complex morphology during the VAs showed a left bundle branch block pattern. Typical twelve-lead ECG of QRS complexes for premature ventricular contractions originating from different portions of TA are documented in Fig. 4.

An rS pattern was recorded in lead V1 in 97.1% (33/34) of patients with VAs from the free wall of TA, vs 2.9% (2/70) of patients with VAs from the septal TA, whereas a QS or Qr pattern in lead V1 occurred in 97.1% (68/70) of patients with VAs from the septal TA vs 2.9% (1/34) of patients with VAs from of the TA. The rS or QS/Qr pattern in V1 was found to be accurate in distinguishing VAs from the septal or the free wall in 97.1% (n = 101) cases.

Among the VAs arising from the vicinity of TA, those arising from the septal portion of the annulus had a shorter QRS duration than the VAs arising from the free-wall portion of the annulus (125.3 ± 5.8 ms versus 149.7 ± 7.2 ms, *P* = 0.001). Furthermore, “notching” of the mid or late component of the QRS complex was observed more often in the VAs arising from the free-wall portion of TA (52.9%) than in those arising from the septal portion of the annulus (Table 2). The precordial R wave transition occurred earlier in patients with VAs originating from the septal portion of the TA (2.8 ± 0.7), than patients with VAs from the free wall (3.5 ± 0.6).

A positive QRS polarity in inferior lead II and aVF was recorded in most VAs with para-Hisian origin (lead II 41/42, aVF 21/42). As for VAs from 3 o’clock and 9 o’clock, a positive QRS polarity in inferior lead II was recorded in 87.5% (7/8) patients. As for VAs from 12 o’clock, a positive QRS wave polarity in leads II, III and aVF was recorded in all patients. As for VAs from 4 o’clock and 8 o’clock, a negative QRS polarity in leads II, III and aVF was recorded in all patients. As for VAs from 5 o’clock and 7 o’clock, a negative QRS wave polarity in leads II, III and aVF was recorded in most (30/32) patients. As for VAs from 6 o’clock, a negative QRS wave polarity in leads II, III and aVF was recorded in all patients.

### Mapping and ablation

Three-dimensional electroanatomical mapping was performed in 92 (88.5%) patients. VTs were induced in 12 patients (11.5%). 70 (67.3%) patients had VAs originating from the septal portion (1 to 6 o’clock region) of the TA and 34 (32.7%) patients from the free wall portion (7 to 12 o’clock region). A detailed distribution of origin of VAs is shown in Fig. 3. During pace mapping, a perfect match of QRS morphology was obtained in 87 (83.7%) patients. In ventricular voltage mapping, none of the patients showed a low ventricular voltage area with a voltage < 1.5 mV, which would suggest a damaged myocardium. The local ventricular activation time recorded at the successful ablation site preceded the onset of the QRS complex by 20.2 ± 4.6 ms. Bipolar pacing was performed at an output just greater than the diastolic threshold from the distal electrode pair during sinus rhythm. Fractionated electrograms were recorded at 85.7% of successful ablation sites. The local electrograms at the successful ablation sites fulfilled the criteria for AV annulus in all patients.

Conventional methods achieved acute success in 53 patients, while reversed U-curve ablation technique eliminated VAs in 43 patients with an overall acute success rate of 92.3%. There were still 8 patients with unsuccessful RFCA by both conventional and reversed U-curve ablation technique, including 2 recurred cases with para-hisian origin. A 3.5-mm irrigated-tip electrode was used in 97.7% (42/43) patients when the reversed U-curve ablation technique was applied. Postulated origins of the failure patients include para-hisian region (5 patients, cessation of ablation because of the appearance of the junctional rhythm and then para-hisian region of the left ventricle was also mapped but no patient was ablated successfully) and inferior septal region (2 patients, inferior septal of left ventricle was also mapped but no patient was ablated successfully). In one patient, procedure failure was observed because of acute cardiac tamponade when mapping. No permanent AV block or other severe complications were seen during or after the procedures.

In 104 patients with frequent PVCs before ablation, 98 (94.2%) achieved acute success after ablation. Two cases with VAs from para-hisian recurred after one month, another two cases from para-hisian origin and one case from 12 o’clock recurred after one year, two cases from 5 and 7 o’clock respectively recurred after more than 2 years. Among these recurrent cases, 5 patients underwent second ablation successfully. Three recurred cases with 5, 7 and 12 o’clock origins were performed repeat ablation through a reversed U-curve ablation technique and irrigated-tip electrode enhanced by a Swartz introducer sheath.

### Complications and follow-up

The final success rate of all 104 patients was 92.3% (96/104) after 35.2 ± 19.6 months (range 3.4–63.9 months) follow-up. Acute cardiac tamponade occurred in one patient and pericardiopuncture was taken. No stroke, coronary arterial damage, or damage of the tricuspid valve was seen in any case. In addition, all 96 patients were free of arrhythmias without antiarrhythmic drugs.

## Discussion

### Main findings

Three major findings were obtained in the present study. Firstly, we reported an algorithm to localize the ablation sites of VAs from the vicinity of TA according to the ECG adjusted by the angle between the cardiac long axis and the horizon. Secondly, fractionated electrograms, the magnitudes of the local atrial and a/V were found to be critical for successful ablation. Finally, we demonstrated that RF catheter ablation was effective for VAs arising from the free-wall portion of the annulus, but its efficacy was a little bit limited for VAs arising from the septal portion. With the reversed U-curve ablation technique, the total success rate reached to 92.3% in our present study which was higher compared to the previous studies^10,11^.

### ECG analysis

A monophasic R or Rs pattern in leadsV5-V6, which showed a left bundle branch block pattern were recorded in all patients with VAs originating from the vicinity of TA. A positive component (any r or R) was recorded in lead I and aVL in almost all patients.

A rS pattern was recorded in lead V1 in 97.1% of patients with VAs from the free wall of TA, whereas a QS or Qr pattern in lead V1 occurred in 97.1% of patients with VAs from the septal portion. To discriminate septal and the free wall origins according to the V1 morphology, the total accuracy was 97.1% (101/104). The precordial R wave transition occurred by lead V3 or earlier in 77.1% (54/70) patients with VAs originating from the septal portion of the TA, as compared to transition beyond V3 in 94.1% (32/34) patients with VAs from the free wall of TA.

**Figure 5:**
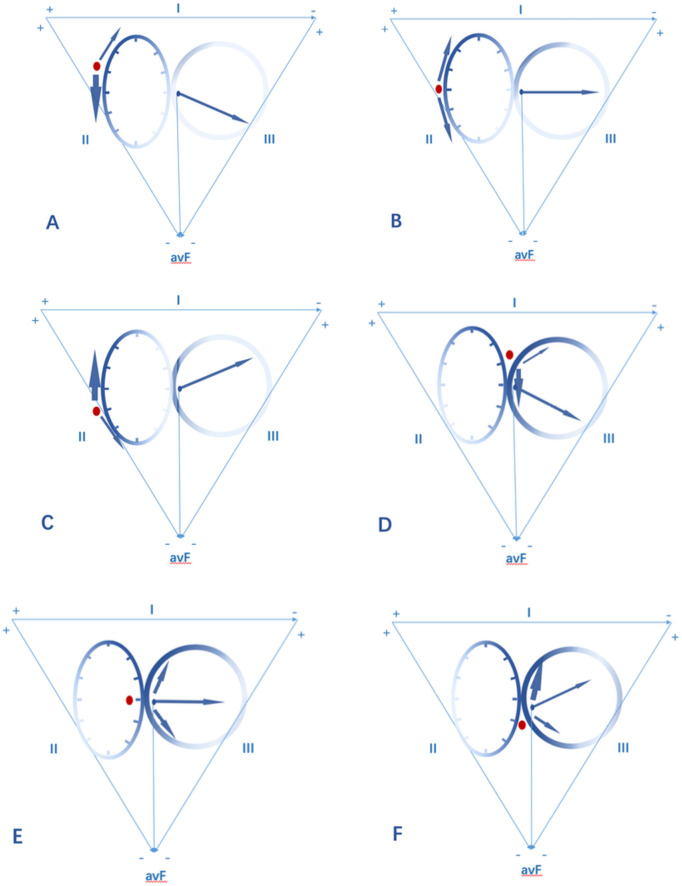
Localization algorithm according to inferior leads II, III, aVF: In the LAO view, TA was viewed as a clock face and the conducting integrated vector of VAs from different positions of TA was showed. (**A**) VAs from 10 o’clock. (**B**) VAs from 9 o’clock. (**C**) VAs from 8 o’clock. (**D**) VAs from para His (1–2 o’clock). (**E**) VAs from 3 o’clock. (**F**) VAs from 4 o’clock. The integrated vector is nearly perpendicular to the direction of lead III, so a rs is seen in lead III (r = s) for (**A**) and (**D**); The integrated vector is nearly perpendicular to the direction of lead aVF, so a rs pattern is seen in lead aVF (r = s) for (**B**) and (**E**); The integrated vector is nearly perpendicular to the direction of lead II, so a rs pattern is seen in lead II (r = s) for (**C**) and (**F**).

After distinguishing the origins of VAs from the free wall or the septal portion of TA according to the V1 morphology and precordial R wave transition, we set up a new algorithm to localize the origins of VAs referred to the QRS patterns in inferior leads. In the LAO view, TA was viewed as a clock face and the conducting integrated vector of VAs from different positions of TA was showed in Fig. 5. For VAs from 10 o’clock, the integrated vector is nearly perpendicular to the direction of lead III. A rs is seen in lead III (r = s) while Rs pattern is seen in Lead II and aVF. VAs higher than 10 o’clock from the freewall always have a positive QRS in leads II, III and aVF. A rS pattern is showed in lead V1; For VAs from 9 o’clock and 3 o’clock, the integrated vector is nearly perpendicular to the direction of lead aVF. A rs pattern is seen in lead aVF (r = s). Rs pattern is seen in Lead II and rS is in aVF. A rS and QS pattern are showed in lead V1respectively; For VAs from 8 o’clock and 4 o’clock, the integrated vector is nearly perpendicular to the direction of lead II. A rs pattern is seen in lead II (r = s). A rS pattern is seen in Lead III and aVF. VAs lower than 8 o’clock from the freewall or 4 o’clock from the septal always have a negative QRS in leads II, III and aVF. A rS and QS pattern is showed in lead V1 respectively; For VAs from para-hisian (1–2 o’clock). The integrated vector is nearly perpendicular to the direction of lead III. A rs pattern is seen in lead III (r = s). A Rs pattern is seen in Lead II and aVF. VAs higher than 2 o’clock from the septal always have a positive QRS in leads II, III and aVF. A QS pattern is showed in lead V1.

According to the QRS pattern of inferior leads, only 44 cases (42.3%) met with the final ablation sites. Among all inaccurate predictions from ECG analysis (n = 60), higher locations were predicted in 12 cases (11.5%), and lower locations in 48 cases (46.2%). It was presumed that there maybe a close relationship with BMI and cardiac transposition. As a result, BMI, the cardio-thoracic ratio, as well as the angle between cardiac long axis and horizon were analyzed as correction factors (Table 3). ECG analysis adjusted by BMI was found to be accurate in predicting the exact ablation sites in 70.2% (n = 73) cases, while the accuracy reached to 92.3% (96/104) when ECG adjusted by the angle between cardiac long axis and horizon. Multiple logistic regression analysis showed that the angle between cardiac long axis and horizon was a critical factor (*P* = 0.009, 95% confidence interval 0.073–0.519). The difference between the predicted and final origins was analyzed and Linear regression was made with the angle between the cardiac long axis and the horizon. The R-square was 30.5% and the coefficient was 0.117 (*P* < 0.01). That is to say, the actual origin was 0.117 o’clock lower than the predicted position by every degree increase in the angle. The final algorithm we used to localize the ablation sites of VAs from the vicinity of TA according to the ECG adjusted by the angle between the cardiac long axis and the horizon was illuminated in Fig. 1.

**Table 3:** Ablation sites predicted on surface ECG after correction.

	Correct position	Incorrect position	*P*
BMI	22.9 ± 1.6	24.4 ± 4.1	0.001
Cardiothoracic ratio	0.43 ± 0.03	0.45 ± 0.04	0.017
Degree of angle*	44.7 ± 1.8	42.1 ± 7.6	0.001

*Angle between the long axis of heart and the horizon in the chest X-Ray.

### Mapping, electrograms and ablation

In these 104 patients, RF energy was delivered at the site where earliest ventricular activation was recorded. For radiofrequency ablation, the acute successful rate was 94.2% (98/104); 4 cases (4/104) recurred around one month after the procedure, and underwent repeated ablation successfully. The local ventricular activation time recorded at the successful ablation site preceded the onset of the QRS complex by 20.2 ± 4.6 ms and by 8.9 ± 3.3 ms in unsuccessful ablation site group (*P* = 0.077).

**Figure 6:**
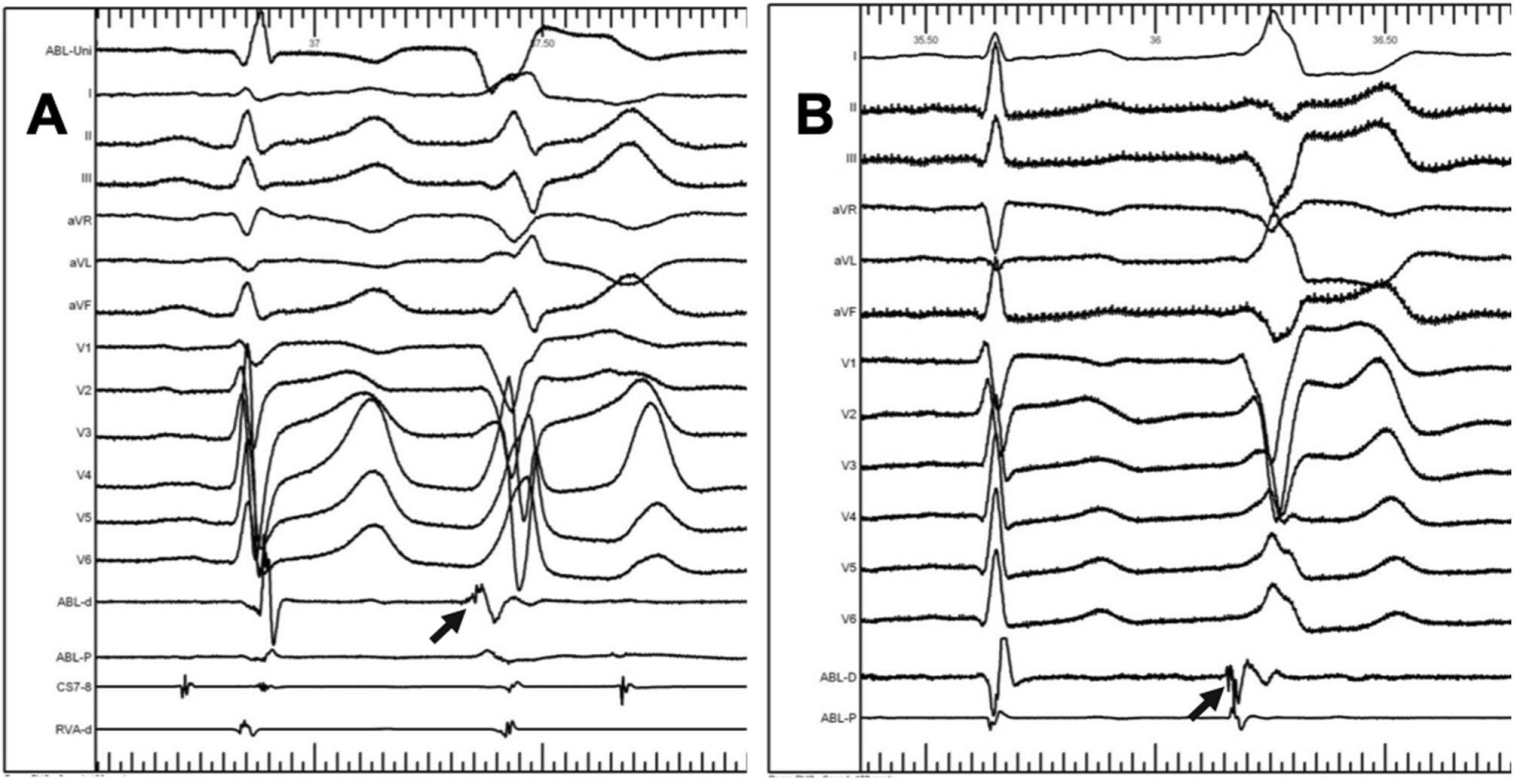
Fractionated electrograms recorded before ventricular electrograms at or around the successful ablation sites. (**A**) VA from 2 o’clock, fractionated electrograms recorded at the successful ablation site preceded the onset of the QRS complex by 20 ms without “a” during sinus rhythm; (**B**) VA from 8 o’clock, fractionated electrograms recorded at the successful ablation site preceded the onset of the QRS complex by 16 ms with “a” during sinus rhythm.

For all the cases, the electrocardiograms of successful ablation sites and invalid ablation sites were both analyzed (Table 4). Fractionated electrograms were recorded at or around the successful ablation sites in 85.7% cases (168/196) while only recorded at 9.5% (36/380) unsuccessful ablation sites during sinus rhythm (*P* = 0.001). At the effective ablation sites, the amplitude of fractionated electrograms is 0.15 ± 0.15mv, and the time of fractionated electrograms is 10 ± 2.9 ms. Fractionated electrograms recorded before ventricular electrograms at or around the successful ablation sites of two cases were shown in Fig. 6. In one case that VA originating from 2 o’clock, fractionated electrograms recorded at the successful ablation site preceded the onset of the QRS complex by 20 ms without “a” during sinus rhythm; while in another case that VA arose from 8 o’clock, fractionated electrograms recorded at the successful ablation site preceded the onset of the QRS complex by 16 ms with “a” during sinus rhythm, which suggested the atrial electrograms “a” might not be indispensable. The magnitudes of the local atrial and ventricular electrograms at the successful ablation site during sinus rhythm were 0.10 ± 0.11 mV (range 0.03–0.47 mV) and 1.62 ± 0.92 mV (range 0.40–3.76 mV) respectively, while the magnitudes of the local atrial and ventricular electrograms at the unsuccessful ablation site were 0.04 ± 0.06 mV (*P* = 0.008) and 1.53 ± 0.83 mV (*P* = 0.255). Binary logistic regression analysis showed that fractionated electrograms, the magnitudes of the local atrial electrograms and a/V ratio were independent risk factors for successful ablation (*P* < 0.05, 95% confidence intervals were 0.002–0.036 and 3.521–7.405 respectively).

**Table 4:** The electrocardiograms of successful ablation sites and invalid ablation sites.

	Successful ablation site	Unsuccessful ablation site	*P*
Earlier than QRS	20.2 ± 4.6	8.9 ± 3.3	0.077
Fractionated electrograms	168/196(85.7%)	36/380(9.5%)	0.001
magnitudes of atrial electrograms (a)	0.10 ± 0.11	0.04 ± 0.06	0.008
magnitudes of ventricular electrograms (V)	1.62 ± 0.92	1.53 ± 0.83	0.255
a/V ratio	0.11 ± 0.18	0.04 ± 0.08	0.001

### Reversed U-curve ablation technique

The reversed U-curve ablation technique is widely used in the RFCA of accessory pathways. It is also suitable for ventricular arrhythmias originating from the right and anterior region of the pulmonary sinus cusp^12,13^. In this study, the conventional method eliminated VAs in only 53 (50.1%) patients while the reversed U-curve ablation technique was conducted to obtain satisfactory contact. However, this technique was feasible in few patients with VAs from the superior septal region especially the para-hisian region because the space between septal cusps of the TA and septal wall of the right ventricle was extremely narrow. The pacing technique^14^ or low energy bipolar ablation^15^ may improve the efficacy and safety for ablation in this region although there is little experience in our institution. An irrigated-tip electrode is extremely necessary for energy delivery in procedures with the reversed U-curve ablation technique because of tight contact.

## Limitations

This study has several limitations. First, this study may be criticized for the relatively small sample size in a single-center and retrospective design without randomization on efficacy of the reversed U-curve ablation technique. Otherwise, irrigation catheter with contact-force-sensing and deflectable sheath was not used in this study. Secondly, our data may not have completely excluded the possibility of a concealed form of arrhythmogenic right ventricular cardiomyopathy. In the present study, although no ECG or echocardiographic abnormalities suggestive of arrhythmogenic right ventricular cardiomyopathy were found, electroanatomical mapping with merged images from CT and MRI, which is useful for diagnosing arrhythmogenic right ventricular cardiomyopathy was not performed. However, the successful ablation indicated accurate mapping.

## Conclusions

The novel ECG algorism adjusted by the angle between the cardiac long axis and the horizon improved the predicted accuracy of ventricular arrhythmias originating from the vicinity of the tricuspid annulus. Fractionated electrograms, the magnitudes of the local atrial electrograms and a/V were demonstrated to be independent risk factors for successful ablation.

## Data Availability

The datasets generated and analysed during the current study are available from the corresponding author on reasonable request.
